# Bees in the city: Findings from a scoping review and recommendations for urban planning

**DOI:** 10.1007/s13280-024-02028-1

**Published:** 2024-05-20

**Authors:** Rutger Remmers, Niki Frantzeskaki

**Affiliations:** 1 Department of Biology, School of Science, Utrecht University, Utrecht, The Netherlands; 2 Department of Human Geography and Spatial Planning, Faculty of Geosciences, Utrecht University, Vening Meinesz building A, Princetonlaan 8a, 3584 CB Utrecht, The Netherlands

**Keywords:** Bees, Biodiversity, Cities, Green space, Pollination, Urban

## Abstract

Over the last decades, bee biodiversity has dropped sharply due to land use change, including urbanization. To contrast this, recent research has pointed to cities as a hotspot for bees. Because of this ambiguity, a scoping review has been conducted to examine the urban characteristics that impact bees and how bees are impacted. A total of 276 articles were analyzed against landscape and local habitat characteristics. The key findings include first that natural areas are more valuable for bees since biodiversity levels are higher. Second, urban areas generally score better than agricultural and rural areas. Third, plant biodiversity positively influences bee biodiversity. Fourth, the urban environment strongly affects some bee traits and the proportion of native bees. For making cities bee friendly and bee inclusive, we recommend to maintain natural areas, connect natural areas to urban ecosystems, encourage floral abundance and diversity and increasing the size of urban green areas overall.

**Supplementary Information:**

The online version contains supplementary material available at 10.1007/s13280-024-02028-1.

## Introduction

As bee biodiversity rates are dropping sharply, cities are taking steps toward becoming bee-inclusive internationally (Marshman and Knezevic 2021). Enhancing bee biodiversity is especially interesting for cities due to the perceived paradox of the city as a refuge for bees and the city as a driver of land-use change and fragmentation (Harrison and Winfree 2015; Glaum et al. 2017; Hall et al. 2017). Humans depend on bees for most of global pollination, an ecosystem service of major relevance to food security, but bees also contribute to other Sustainable Development Goals (Willmer et al. 2017; Patel et al. 2021). Unfortunately, there is no panacea for increasing urban bee biodiversity, and methods are often limited to basic steps, such as increasing floral abundance and richness (Threlfall et al. 2017; Turo and Gardiner 2019).

Within urban bee ecology, a relatively new field, research is taken up that must aid bee conservation in the city (Hernandez et al. 2009). Going beyond floral measures, urban bee ecology may inform urban planning for the design of inherently bee-friendly cities. Consequently, for cities to become bee inclusive, it is important to determine how the urban environment and its characteristics impact these pollinators. Numerous researchers are hypothesizing on measures that need to be taken to balance out bee communities, as well as specific traits (Buchholz and Egerer 2020; Ferrari and Polidori 2022). To counter the effects of urbanization within the city’s perimeter, a more systematic examination and knowledge synthesis is required about the effects of urbanization and urban areas on bee biodiversity.

Evidently, cities act as a filter for bees, resulting in urban “winners and losers” (Banaszak-Cibicka and Żmihorski 2012). Beyond biodiversity indices, cities also influence the traits of bees that are frequently encountered (Buchholz and Egerer 2020). Which bee traits are functionally relevant within urban bee ecology is not set in stone; some researchers include more bee traits than others or may order traits differently. For example, Williams et al. (2010) distinguish body size (continuous), nesting behavior, nest construction, trophic specialization (lecty, diet), and sociality. Contrarily, Twerd and Banaszak-Cibicka (2019) note body size, nesting behavior, trophic specialization, and sociality including cleptoparasitic. Other aspects include tongue length, emergence during the bee season and temperature breadth (Buchholz and Egerer 2020; Ayers et al. 2021).

Another aspect of bees that is frequently mentioned is the native status of bees, which researchers sometimes treat as a trait of equal value to biological traits. Due to the abundance of exotic, ornamental plants in urban areas, exotic bees are also attracted to urban areas (e.g., Molumby and Przybylowicz 2012; Salisbury et al. 2015). Furthermore, urban beekeeping is becoming increasingly popular (Egerer and Kowarik 2020). Consequently, the proportion of exotic bees in cities, specifically honeybees is relatively high. Though not all honeybees are managed by beekeepers (Youngsteadt et al. 2015), some researchers prefer to exclude *Apis mellifera* L. altogether (e.g., Choate et al. 2018; Buchholz et al. 2020; Kammerer et al. 2021). For other bees, the city’s geography would determine whether a bee is exotic or not.

Below-ground nesting bees need bare soil to nest, so they are theoretically negatively affected by impervious surfaces (Matteson et al. 2008; Fortel et al. 2014). However, not all traits are predicted easily, and research literature is contradictory to this matter (Buchholz and Egerer 2020). For example, large bees are expected to be advantaged, as their size allows them to forage further. The downside is that larger bees also require more energy, and it remains disputed which effect is stronger (Ferrari and Polidori 2022). Consequently, it may be difficult for cities to plan for bee biodiversity. This paper will, therefore, attempt to answer the following overarching research question: How do bees respond to urban landscape characteristics? Research sub-questions aiding our analysis are: which urban characteristics benefit bees in cities? What are the knowledge gaps in the current state of the art that need further examination to make urban environments bee-inclusive and bee-friendly?

Against this background, we conduct a scoping literature review in accordance with Arksey and O’Malley (2007), to summarize and extend the analysis to actionable knowledge for urban design and urban planning to support bee-inclusive and bee-friendly cities. Four perspectives will be taken regarding bee biodiversity, namely, urban environment, local urban habitat, green roofs, and housing. The former three are clear themes from research literature and are at the cross section of urban planning and urban bee ecology. Though relatively new to the mix, green roofs are already fully taken up in such research (Rahimi et al. 2022). Bee housing is also included due to its accessibility as a measure to cities (MacIvor and Packer 2015; Rahimi et al. 2021). In this review, honeybees will also be included as they are undeniably part of the city’s bee composition.

## Methodology

A review was conducted to investigate how bees are affected by urban environments at both the local and the landscape scale. The goal of this review is to synthesize an overview of state of the art on which urban characteristics benefit bees in cities and to find knowledge gaps in literature that need further examination, using the framework for scoping reviews given by Arksey and O’Malley (2007). The review is conducted following the following subsequent steps (Table 1).

**Table 1:** Scoping review steps and process (*Source*: Authors)

Steps	Procedure	Accepted articles
1. Data gathering	Web of Science and Scopus search, through the following search string: “TITLE-ABS-KEY (bee OR apidae AND urban* OR city OR roof AND NOT algorithm)”	1397
2. Data screening and cleaning	Title reading with the following criteria: Are bees involved? Is an urban component present?	834
	Abstract reading following the bellow screening criteria (exclusion): Articles on bees as biomarkers were excluded Articles on beekeeping practices were excluded Articles with no evidence of the ability of bees to pollinate were excluded Articles with no reference to urban environment were excluded To add to these criteria, the papers were double-screened through replying to the following analytical questions: Is the urban component weighed? Are bees researched for aspects related to their ability to pollinate?	499
3. Data scoping	All available full texts were downloaded	460
4. Article appraisal and analysis	Full text reading using the criteria from step 3	276

### Data gathering

Scopus and Web of Science were used to identify a list of articles related to the topic. The literature search was applied in both search engines for their elaborate range of interdisciplinary research. The articles were retrieved at the beginning of December 2022. No articles were excluded based on their publication date. The initial query produced a collection of 2179 articles, 782 of which were excluded immediately for being duplicates. The search query was: “TITLE-ABS-KEY (bee OR apidae AND urban* OR city OR roof AND NOT algorithm)”. Its functional equivalent was used in Web of Science.

### Data screening

The remaining 1397 articles were screened in three steps. In each step, articles were excluded if their relationship to bees and urban form was not sufficiently present. This was performed in three steps to guarantee that exclusion was done cautiously. During abstract reading, articles were checked for comparisons between two locations or along an urban gradient to determine the effect of the urban environment (urban weighing).

### Article appraisal and analysis

During data extraction, the steps of data screening on abstracts were now applied to full texts, during which another 183 out of 460 articles were eliminated. Articles that involved more than only bees (e.g., Lepidoptera, Syrphidae) were also included on the condition that results on bees could be distinguished from the data. The remaining data were chartered into subtopics and were used for critical analysis. Charting of the 276 articles was performed using four analytical themes: landscape characterization, habitat characterization, green roofs, and housing. The results were grouped among six themes determined a priori: biodiversity and survival, native status, size, sociality, nesting, and lecty. During data collection, results were clustered when insufficient results were obtained for multiple specific factors, but their combination would not lose specificity to said factors. These include colony dynamics, pollen collection, varroa load, parasitism, and the honeybee effect (Supplementary data). For biodiversity indices, composition, and evenness are clustered under diversity, and density is clustered with abundance. For all articles, the following information was also noted: goal, species, output measures, geography, and, if present, policy recommendations. A table of the analysis of the 276 articles is included as Supplementary Material detailing the screening and mapping of the selected records against the analytical framework.

Papers on landscape characteristics apply larger radii than papers on habitat characteristics. The former use radii between 200 and 1500 m, often derived from the foraging distances of the bee in question. Habitat characteristics are determined in a smaller area, e.g., a plot of 100 m^2^. Papers on landscape characteristics commonly include the inner circle that would also be used to research habitat characteristics, thus including both areas.

For landscape characterization, urban areas were compared to other habitats, which include natural, agricultural, suburban, and rural categorization. Articles were consistently followed in their categorization. Alternatives to categorization, such as % impervious surface, were also followed consistently. These were clustered as general urban comparisons. Some articles compared multiple areas (e.g., urban versus agricultural and urban versus natural). These were both entered separately, as these domains are also analyzed separately.

For habitat characterization, factors influencing bee traits and three biodiversity outcomes (abundance, richness, alpha diversity) were determined before and during the data extraction. Factors determined beforehand were floral factors (richness, abundance, diversity), isolation, area size, the native status of vegetation, buildings, and ecomanagement. Additionally, several habitat types were included. During analysis, only factors that contained at least 3 hits were included.

After data gathering and careful screening, 276 articles were analyzed for various properties. These include bee species, geography, and properties related to landscape characterization, habitat characterization, green roofs, and housing. 225 of 276 articles concerned the urban environment, followed by 78 on habitat characterization. Only 8 articles were on green roofs, and 5 articles were on housing as urban characteristics impacting bees. Therefore, no analysis was performed on these themes.

## Results

### Charting the results

The review of the selected records shows that the most investigated species concerned *A. mellifera* (51), in part due to papers on the relationship between apiaries and their location. However, most articles (162) considered entire bee communities, in aim of providing a comprehensive overview of the species found in urban and non-urban areas. Furthermore, articles focused specifically on bees with certain traits (13), stingless bees (18), *Bombus spp.* (51), wild bees (16) and native bees (6). In the latter two, *A. mellifera* is specifically excluded (or assumed to be). These articles did not concern apiaries. Excluding *A. mellifera*, 47 articles were on one species and 10 articles were on multiple specific species not bound by traits or genus. Notably, for further analysis of the results, all species are pooled.

We also found that the geography of research results is predominately in the northern hemisphere with 112 articles (from the 276), reporting on research performed in the North America. Europe produced close to the same amount of research records (105) with most papers from Germany (25), England (16), Poland (14) and France (13). Much less articles are produced in South America and Oceania, with, respectively, Brazil and Australia dominating in these areas. Like other recently published review studies (Brant et al. 2022; Prendergast et al. 2022a, b), it is evident that research efforts focus on developed countries in North America, Western Europe, and Australia. Only a few papers draw on cross-case study and cross-country results.

### Which landscapes are better for bees: Natural, urban, sub-urban, or agricultural?

Table 2 shows the number of positive, negative, and neutral remarks of each habitat type, versus the urban habitat. For example, the 7 positive (“+”) remarks in the “Natural”-section on abundance show that 7 articles were found that show that urban areas have a higher abundance of bees compared to natural areas. On the contrary, 13 negative (“−”) remarks conclude that natural areas perform better than urban areas concerning bee abundance.

**Table 2:**
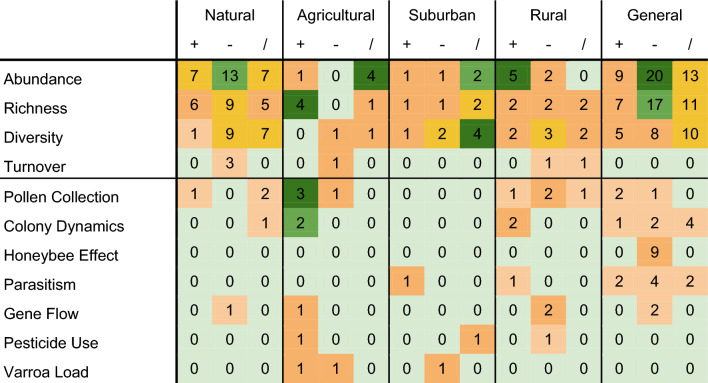
Number of remarks on biodiversity indices and other measures for habitat comparisons and general comparisons. “+” = Urban better than …; “−” = Urban worse than…; “/” = No significant difference between urban and …. The colors indicate the weight of the number of remarks, relative to its habitat or column. Gray green indicates no remarks, light orange indicates between 0 and 5%, dark orange indicates between 5 and 10%, yellow indicates between 10 and 15%, light-green indicates between 15 and 20%, dark-green indicates 20% or higher

Also, clear from other indices, natural areas are more suited for bees than urban areas. For example, Abbate et al. (2019) show that the abundance and richness of bees are higher in protected lands than in developed sites. The abundance of bees is generally higher in natural areas, for example, *Bombus* species (Schochet et al. 2016). The same pattern is visible for richness and diversity, each of which is generally higher in natural areas. The high number of positive and neutral results nonetheless demonstrate inconclusiveness. Despite this discrepancy, the differences between natural and urban areas explicitly favor the former. This is further supported by species turnover that is higher in natural areas, depicted by three negative remarks (Verboven et al. 2014; Hung et al. 2017; Harrison et al. 2018b).

Urban areas are likely more suited for bees than agricultural areas. In contrast to the urban-to-natural comparison, the urban-to-agricultural comparison shows a distinction between the abundance and richness of bees. Abundance is unaffected in the urban environment, resulting in, for example, a similar number of visits per plant species, as shown by Geslin et al. (2013. The higher bee richness in urban areas in comparison with agricultural areas reflects the more diverse supply of food and possibly nesting locations (Baldock et al. 2015; Theodorou et al. 2017). Bee diversity, however, is lower in urban areas compared to agricultural areas, though both Harrison et al. (2018a) and Christman et al. (2022) expect the intensification of agriculture to affect negatively. Pollen collection is also higher in urban areas (Alburaki et al. 2018; Brodschneider et al. 2021; Renaud et al. 2022).

Due to only a few comparisons between urban and suburban areas, no strongly convincing correlations are found. There are relatively many neutral mentions for bee abundance, richness, and diversity, up to 50% per index. Amado De Santis and Chacoff (2020) found that bee diversity and richness are very comparable between urban and suburban sites in the biodiverse region of Yungas, Argentina, for example. In rural areas, abundance is evidently lower than in urban areas. Richness and diversity show no direction in which bees are advantaged, as also shown by Zaninotto et al. (2021). It can be concluded that rural areas contain fewer bees, but a similar number of bee species compared to urban areas.

Finally, based on papers using general urban measurements making use of %impervious or other urban gradients, it becomes evident that urban areas have lower bee abundance and richness. For example, Pereira et al. (2021) show that bee richness has decreased over 60 years in southern Brazil. Notably, the number of studies that found positive or neutral results is also high, respectively, above 5% and 10%. Positive effects are sometimes attributed to a more diverse landscape (Egerer et al. 2017; Martins et al. 2017) though the opposite also has been found (Janvier et al. 2022). Diversity, however, is met with less negative results, showing diversity can be somewhat maintained in urban areas. Colony dynamics appear unaffected by urban landscape characteristics, with 4 neutral remarks, against 1 positive and 2 negative remarks. The honeybee effect, a direct influence of the presence of honeybees on bee composition, is noted 9 times.

### Bee traits: Which bees thrive in urban environments, native or exotic, small or big?

The urban environment strongly affects some bee traits and the proportion of native bees. Urban areas contained more exotic bees (Collado et al. 2019; Birdshire et al. 2020; Zaninotto et al. 2020). Out of these cases, half were seen in the USA, 5 in Oceania, and 3 in Europe. For the remaining 7 cases, 3 remarks were made of urban form leading to less exotic bees and 4 cases of the native status being unaffected. For example, Lowenstein et al. (2014) show that the presence of exotic bees is unrelated to human population density, a proxy for urbanization. Notably, some researchers deliberately exclude honeybees from their assessments, thus underrepresenting the number of exotic bees and not representing beekeeping activities (Carré et al. 2009; McCune et al. 2020; Casanelles-Abella et al. 2022). Otherwise, ornamental and exotic plants increase exotic bee species (Threlfall et al. 2015; Sivakoff et al. 2018; Wilson and Jamieson 2019; McCune et al. 2020; Casanelles-Abella et al. 2022).

In most cases, bees become smaller or are unaffected in their size in urban environments. In relatively few papers, the contrary is seen, and bees become larger. It is hypothesized that small bees are advantaged since smaller bees need fewer resources (Banaszak-Cibicka and Żmihorski 2012; Fortel et al. 2014). At the same time, large bees would be favored due to their ability to cover longer foraging ranges (Bennett and Lovell 2019; Ropars et al. 2019). With factors that may benefit small and large bees differently, no conclusion can be drawn on how urban environments affect bee size. This conclusion is further supported by the relatively high number (16) of neutral articles.

Urban environments generally favor cavity-nesting bees, with close to half of the papers showing their abundance rising. The remaining papers show the nesting behavior to be advantaged to ground-nesting or to be unaffected. A major reason for ground-nesting bees to be disadvantaged is the lack of nesting surface in urban areas (Fortel et al. 2014; Threlfall et al. 2015; Pereira et al. 2021). On the contrary, cavity-nesting bees can nest in (abandoned) buildings or other urban cavities (Zanette et al. 2005; Pardee and Philpott 2014; Hamblin et al. 2018).

Trophic specialization is evidently lower in urban areas, thus giving rise to polylactic bees in urban areas. They are generally less sensitive to urbanization, but also do not depend on specific flower species for their foraging (Fetridge et al. 2008; Choate et al. 2018; Geppert et al. 2023). Wray and Elle (2015) note that these primarily concern *A. mellifera*, *Bombus spp.,* and solitary cavity nesters. Larger cities would generally contain more exotic and ornamental plants, limiting resources for oligolectic bees (Ferrari and Polidori 2022).

Finally, eusocial bees are more present in urban areas compared to solitary bees. Notably, there are no strong theories on why which bee type is advantaged (Carper et al. 2014; Guenat et al. 2019; Cohen et al. 2022a, b). Notably, urban landscapes lead to exotic, small, cavity-nesting, generalist, social bees. These traits also fit the description of *A. mellifera*, the honeybee. Though not all articles include honeybees (Fauviau et al. 2022; Graf et al. 2022), it is evident that honeybees and bees with similar traits thrive in urban areas.

### Habitat characteristics

There are relatively few articles that assess both landscape and habitat characteristics. Some articles that do so on biodiversity indices note that landscape factors are not significantly relevant, but habitat characteristics are. This hints at habitat characteristics acting as a substitute for landscape characteristics. Table 3 shows how habitat characteristics affect bee traits and biodiversity within urban areas. Darker and greener colors indicate stronger correlations relative to its column. Notably, some columns have only a few notions and therefore contain higher colors.

**Table 3:**
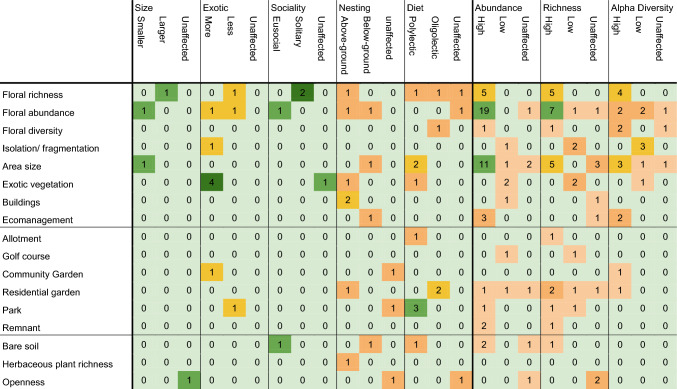
Number of remarks for bee traits, native status and biodiversity indices for several habitat factors and habitat types. The colors indicate the weight of the number of remarks, relative to its habitat or column. Gray green indicates no remarks, light orange indicates between 0 and 4%, dark-orange indicates between 4 and 8%, yellow indicates between 8 and 16%, light-green indicates between 16 and 32%, dark-green indicates 32% or higher

The abundance of bees is primarily increased through floral abundance, area size, and floral richness in said order. The former two are predicted to be relevant since they increase the chance of successful foraging. Stewart et al. (2018) show both are the case for green areas in a tropical megacity in Thailand. Floral richness likely increases bee abundance by increasing bee species richness (Theodorou et al. 2020; Griffiths-Lee et al. 2022). It is probable that floral richness increases bee abundance through bee species richness. Floral additions, through either pathway, remain a safe and consistent way of increasing bee abundance. Few remarks are also made of the positive effects of ecomanagement. This may be expected since less mowing allows for higher abundance of native plants.

Positive effects on species richness can also be found in floral abundance, floral richness, and area size. However, there are cases in which area size has no effect, possibly due to the small size of the investigated habitats (Loyola and Martins 2011; Gunnarsson and Federsel 2014). Isolation and exotic vegetation negatively affect richness. Isolation of areas decreases the total area available to support bees, thus limiting species especially smaller species (Banaszak-Cibicka et al. 2016). Additionally, isolation enlarges the competition between the honeybee and wild bees, favoring the former (Plascencia and Philpott 2017). Similarly, exotic vegetation is generally less attractive to native bees, thus reducing their richness (Molumby and Przybylowicz 2012; Zhang et al. 2022).

Alpha diversity is positively affected by floral richness, floral diversity, area size, and ecomanagement. Most likely, this is due the same reasons as given above for abundance and richness. Reduced mowing showed overall higher biodiversity indices, especially for bee diversity (Wastian et al. 2016). Notably, floral abundance is noted for both increasing and decreasing diversity, as determined by 2 mentions for both. One of its negative remarks points toward a possible interaction with honeybees (Plascencia and Philpott 2017). Like abundance and richness, isolation also negatively affects diversity, with most negative remarks.

Native bees are negatively affected by exotic vegetation, as they increase competition from exotic bees (Molumby and Przybylowicz 2012; Threlfall et al. 2017) and are less suited to native bees (Salisbury et al. 2015; Potter and Mach 2022). Competition by introduced bee species is either achieved directly through limited nesting spaces, limited floral resources or territorial behavior (Russo et al. 2021; Potter and Mach 2022), and can thus affect plant-pollinator networks on an ecosystem level (Geslin et al. 2013).

Too few notions on bee size and sociality were made in papers on habitat characterization. Buildings in the proximity generally favor above-ground nesting bees, a direct consequence of less area available for ground-nesting and more nesting sites for cavity-nesting bees (Zanette et al. 2005; Lanner et al. 2020). Only area size appears to affect diet, with polylectic bees gaining advantage in larger areas (Antonini et al. 2013; Zaninotto and Dajoz 2022). This also fits with their most suited habitat, the park, which is generally larger than other habitats. Residential parks, on the other hand, benefit oligolectic bees, possibly due to a higher plant species richness (Sirohi et al. 2022).

## Discussion

In this scoping review, we investigated the effects of urban form characteristics on bees. From the reviewed records, what represents “urban” and the key characteristic of the urban environment that attracts bees need to be discussed here. Within the urban form, the urban degree is mostly measured through the percentage of impervious surfaces or other urban gradients such as human population density. However, the urban degree is not fixed on such measures and is not uniform across the literature. Some articles compare three degrees of urbanization, though often based on the categorization of urban, suburban, and rural areas (Amado De Santis and Chacoff 2020; Banaszak-Cibicka and Żmihorski 2020; Łoś et al. 2020) or otherwise from inner to outer city (Hülsmann et al. 2015; Theodorou et al. 2022). Though resulting in a more complete picture, clear distinctions between urban, suburban, and rural areas are vital for assessing the effect of urban form on bees. That is something we found requiring closer attention and interdisciplinary collaboration to progress. Similarly, rural and agricultural areas may be used interchangeably for agricultural areas due to the low human population density in found agricultural areas. Instead, in this work, habitats are set off against an urban counterpart. A common thought among researchers is that low degrees of urban form increase biodiversity. In contrast, higher degrees of urban form do not increase biodiversity (Ferrari and Polidori 2022), which is supported only by the comparison of urban and suburban areas.

Comparing our findings with other review articles (see Appendix, Table S.1), we contend that we have presented an overarching analysis to respond to the research question. Our literature review complements other reviews in the field that also focus on bees and pollinators but adds to the field by casting an eye on the landscape characteristics and related aspects that affect urban bees. As visually shown in Table 4, our results agree with previous reviews but are broader and cover more dimensions relevant to examining how urban environment characteristics affect urban bees. In comparison with Wenzel et al. (2020) and Liang et al. (2022), we agree with the findings that empirical data are limited geographically, and as we propose, future research needs to have a global focus. We are also in agreement with Liang et al. (2022) that biotic and abiotic factors play important role in making urban environments bee-friendly, but our review extends to bring our findings to urban planning through a suite of recommendations. In relation to Wenzel et al. (2020), we present that urban characteristics that affect bees are beyond building height (as Liang et al. 2022 note) but also relate to availability of green spaces and their connectivity. Specifically, our review extends to translate findings to urban planning and landscape implications that are contributing to interdisciplinary dialogue between urban ecology and urban planning. This is a unique contribution we are making in this paper, to offer an inter-disciplinary translation of our findings that can inform urban planning and landscape planning actions at strategic and operational levels.

**Table 4:** Recommendations to urban planning for making urban areas bee-inclusive

#	Recommendation	Supporting articles
1.	Focus on habitat characteristics	Kearns and Oliveras (2009), Matteson and Langellotto (2010), Antonini et al. (2013), Williams and Winfree (2013), Pardee and Philpott (2014), Felderhoff et al. (2022), Gerner and Sargent (2022), Persson et al. (2022),
2.	Maintain natural areas and create ‘connections’ between natural areas and urban ecosystems	Hisamatsu and Yamane (2006), Fetridge et al. (2008), Banaszak-Cibicka (2014), Neil et al. (2014), Verboven et al. (2014), Clermont et al. (2015), Wray and Elle (2015), Schochet et al. (2016), Hung et al. (2017, 2019), Cândido et al. (2018), Harrison et al. (2018a, 2018b, 2019), Razo-León et al. (2018), Abbate et al. (2019), Collado et al. (2019), Yasrebi-de Kom et al. (2019), Marín et al. (2020), Wayo et al. (2020), Mráz et al. (2021), Prendergast and Ollerton (2021), Shrestha et al. (2021), de Matos Barbosa et al. (2022), Maher et al. (2022), Prendergast et al. (2022a, b), Renaud et al. (2022), Simla et al. (2022)
3.	Promote floral richness and abundance	*Floral richness* Matteson and Langellotto (2010), Lowenstein et al. (2014), Pardee and Philpott (2014), Quistberg et al. (2016), Stewart et al. (2018), Wilson and Jamieson (2019), Lanner et al. (2020), Theodorou et al. (2020), Novotny et al. (2021), Felderhoff et al. (2022), Gerner and Sargent (2022), Griffiths-Lee et al. (2022), Persson et al. (2022)
		*Floral abundance* Zanette et al. (2005), Ahrné et al. (2009), Matteson and Langellotto (2010), Hennig and Ghazoul (2012), Williams and Winfree (2013), Bennett and Lovell (2014), Pardee and Philpott (2014), Threlfall et al. (2015), Quistberg et al. (2016), Makinson et al. (2017), Stewart et al. (2018), Clos et al. (2020), Daniels et al. (2020), Lanner et al. (2020), Alves and Gaglianone (2021), Berthon et al. (2021), Kanduth et al. (2021), Novotny et al. (2021), Gerner and Sargent (2022), McDougall et al. (2022), Persson et al. (2022)
4.	Plant native vegetation and otherwise limit honeybees	Molumby and Przybylowicz (2012), Salisbury et al. (2015), Threlfall et al. (2015), Martins et al. (2017), Plascencia and Philpott (2017), Ropars et al. (2019), McCune et al. (2020), Meeus et al. (2021), Prendergast et al. (2021), Weissmann et al. (2021), Casanelles-Abella et al. (2022), Patenković et al. (2022), Potter and Mach (2022)
5.	Increase the size of urban green areas	Zanette et al. (2005), Nemesio and Silveira (2007), Matteson and Langellotto (2010), Hennig and Ghazoul (2012), Muratet and Fontaine (2015), Quistberg et al. (2016), Makinson et al. (2017), Michołap et al. (2017), Stewart et al. (2018), Lozier et al. (2020), McCune et al. (2020), Cândido et al. (2021), Turo et al. (2021), Cohen et al. (2022a, b)

A different proxy for urban form that may be useful for bee biodiversity is temperature. Due to the urban heat island effect, higher temperatures are seen in areas with higher urban degrees and therefore, it acts as a proxy. Articles that show temperature is relevant to bee biodiversity and filtering are clustered under urban environments. However, only a few researchers refer to temperature is a driving force (Hamblin et al. 2018; Kammerer et al. 2021; Geppert et al. 2023). Notably, their papers are recent and may prove useful in conservation of bee biodiversity. As pointed out by Banaszak-Cibicka (2014), an increase in temperature results in an emigration up north of Mediterranean bees. Consequently, a different composition in urban areas may be the consequence of migrating bees instead of being a consequence of urbanization. Notably, sunlight is also generally accepted to be a major predictor of bees (Matteson and Langellotto 2010; Everaars et al. 2011; Williams and Winfree 2013). With these in mind, we want to bring two discussion points to our attention: First, are cities friendly or hostile environments to bees? And stemming from this, we propose five recommendations to urban planning (including urban design, ecology, and architecture measures regulated by it) to transform urban environments into bee-friendly and inclusive ones.

### Are cities friendly or hostile environments for bees?

In their review, Prendergast et al. (2022a, b) note that abundance is often higher in urban areas, whereas richness is lower compared to natural areas. That is not what we found in this review, but rather, bee abundance, richness, and diversity are lower in urban areas. However, richness is higher in urban areas, compared to agricultural and rural areas, and so is abundance compared to rural areas. Most likely, this difference is due to the higher diversity of habitats found in the urban landscape. The negative effects of urban areas are also found in the general comparisons, highlighting many more negative remarks than positive remarks (see Table 2). This is further supported by the positive relationship between floral diversity and bee diversity (see Table 3). In conclusion, urban areas are relatively hostile environments for bees when compared to natural areas but friendly environments when compared to agricultural areas.

Bee traits are also affected by urban form. Remarks on bee composition are most likely related traits since some traits are convincingly advantaged in urban areas. However, there appears to be no consistent way in which specific traits are advantaged or disadvantaged. Most clearly, urban areas contain more exotic bees, and with a close second, urban form also benefits generalist bees. Though less evident, urban areas favor smaller, more social, and cavity-nesting bees, though the number of neutral remarks on size and nesting type is high. Notably, research that includes honeybees, which are generally dominant in urban environments, all traits are likely skewed toward honeybees. The honeybee is a small, social, cavity-nesting, and generalist bee. These traits are also advantaged in urban areas, hindering the assessment of what traits are favored in urban areas beyond honeybees. Therefore, it appears urban areas are selectively hostile to bees, thus acting as a filter.

### Planning for urban habitats for bees

Urban areas may host high numbers of bees of various species. Their biodiversity can be supported through various methods, as deduced by the results of habitat characterization. Relatedly, we draw recommendations for urban planning to support bee habitats in the urban environment; or simply to help make cities bee-inclusive and bee friendly. The recommendations are supported by several articles from our review and are listed in Table 4.

The first recommendation is to focus measures on habitat characteristics rather than on landscape characteristics or habitat types for bee conservation. Focusing solely on landscape characteristics does not aid in bee diversity, which is the primary goal of urban bee ecology. Several articles show that habitat type is not relevant to biodiversity indices. Rather, landscape characteristics and habitat types may hint at where higher biodiversity of bees can be found. Notably, habitat characteristics make up habitats and landscape at larger scales. For example, an area with high floral richness and abundance is more likely to be an unmanaged natural area than a highly urbanized area. Focusing on improving habitat characteristics also allows practitioners to include urban areas in their work.

Second, maintaining natural areas is an important measure for protecting bee biodiversity. Natural areas are hotspots for bees that can maintain themselves independently. Notably, natural areas are more suited for bees than urban areas are. This way bee biodiversity is maintained, while giving time to cities to make their cities more bee friendly. Natural areas close to urban areas or connections between natural areas and urban ecosystems may be the measures to populate cities with bees in the future.

Third, in cities, floral richness and floral abundance need to be promoted and managed/maintained. Increasing floral richness and floral abundance also positively affects bee abundance, richness, and diversity. Improving these aspects is a relatively easy and cost-effective way of nurturing bee biodiversity. Research has also found that involving stakeholders in conservation practices may increase their effectiveness, especially when greenery is involved, something we have seen over the years in several cities involving children, youth, the elderly and the general public in flower bed plantings (Hall et al. 2017; Braman and Griffin 2022; Brom et al. 2022).

Fourth, measures to plant native vegetation need to be prioritized and need to come hand in hand with measures to limit beekeeping. Specifically, promoting native vegetation, specifically vegetation that suits oligolectic species, may advantage native bees and contribute to reducing the proportion of exotic bees in urban areas (by lowering in this way competition for native bees). This applies to both public areas especially urban green areas and private green areas, though for the latter, tailored campaigns and incentives for native greening may be required. Honeybees are a major competitor in urban areas, in part due to beekeeping. Limiting beekeeping would prove effective in limiting competition with exotic species. However, interventions may affect commercial parties and hobbyists, thereby require careful assessment and collaborative planning approaches.

Fifth, increasing the size of urban green areas has proven to be an effective measure to promote bee biodiversity overall. In this review, it has come to light that area size is related to bee abundance, richness, and diversity. It may be particularly difficult in dense cities to carry out this measure due to a lack of space. In such cases, connecting existing green spaces may be valuable first steps. Nevertheless, in line with the second recommendation, (near-)natural areas are invaluable for maintaining bee biodiversity. Efforts must therefore be made to increase green area sizes in urban and peri-urban, rural areas.

### Knowledge gaps and directions for future research

Our review identified a number of knowledge gaps listed below that can inform future research topics:

- *Geography of research and evidence*: Looking at the geography of the conducted research, it becomes immediately clear that disproportionally few articles from Africa, Asia, and South America are available. Moreover, the ecology of these areas is different from that of the Western world. Combined, it becomes evident that research from North America, Europe, and Australia cannot be directly projected onto these areas. Thus, to make conservation practices relevant locally, future research must also involve these areas.
- *Species turnover*: Another gap in research identified by this review concerns the effect on urban areas on species turnover. Despite researching multiple areas for comparison, turnover is often unmentioned. In this review, it was noted only six times. Since urban areas are generally seen as filters to bees, beta diversity should be deemed an important index for its complete understanding.
- *Bee housing*: There were too few papers on bee housing and green roofs to include these. More research on these topics would allow for systematic analysis of their effects. Since bee housing is an accessible manner of increasing bee biodiversity, it would be valuable to determine their effectiveness. Valuable reviews on housing and green roofs have been performed by Hofmann and Renner (2018) and Rahimi et al. (2021), respectively.
- *Local habitat characteristics*: Literature on local habitat characteristics may make research on the effect of habitat types of biodiversity redundant. Insights into these characteristics can also be applied to all habitat types. Related to this, another knowledge gap concerns how traits are affected by local habitat characteristics. Future research is required to go further than floral indices, with inclusion of local plant composition that advantage native bees (Filipiak 2019; Nichols et al. 2019).
- *Best urban design and urban landscape planning practices for bees and pollinators*: With the reviewed literature in mind, we argue that the current state of the art does not provide more specific directions on ‘how’ or what effective urban landscape designs have been tested or can be considered as good practices to this end. We propose that future research needs to work to this end, given that even in urban plans for biodiversity or bees specifically (see for example City of Sydney, Australia on biodiversity strategy or the City of Rotterdam’s biodiversity strategy) there are no specific frameworks to evaluate the effectiveness of urban measures toward pollinator spaces or diversity safeguards. At the same time, even EU Biodiversity Strategy points to the importance of pollinators but does not extend to specifying specific good practices for urban landscape connectivity. The recently under consultation and public debate USAID Biodiversity strategy also acknowledges pollinators but does not extend to specific directions on improving bee-inclusivity and pollinators’ diversity in urban landscapes. Without the aforementioned being a systematic record of what can be considered a policy gap, we can propose that more research is needed to extend our recommendations (Table 4) to urban best practices for bees and other pollinators.

Despite these research gaps, it is evident that local and regional governments must not wait for future research before making interventions. Waiting for the filling of research gaps would only postpone the restoration of bee biodiversity.

### Supplementary Information

Below is the link to the electronic supplementary material.

Supplementary file1 (PDF 474 KB)

Supplementary file2 (XLSX 224 KB)
