# Supplementary material for: Bees in the city: Findings from a scoping review and recommendations for urban planning
Source: Ambio. 2024 May 20;53(9):1281–95. doi: 10.1007/s13280-024-02028-1 (PMC11300792; doi:10.1007/s13280-024-02028-1)
Supplement: Supplementary file 1 — Supplementary file1 (PDF 474 KB) [file 13280_2024_2028_MOESM1_ESM.pdf]

Supplementary Material: Full records screened and included in the review

This is supplementay to the article "**Bees in the city: Findings from a scoping review and recommendations for urban planning**"

Rutger Remmers
Department of Biology, School of Science, Utrecht University, The Netherlands

Niki Frantzeskaki
Department of Human Geography and Spatial Planning, Faculty of Geosciences, Utrecht University, The Netherlands

Keywords: bees, biodiversity, urban, cities, pollination, green space

Conflict of Interest Statement
Authors declare no conflict of interest.

Appendix: Table S.1. Juxtaposition of review articles on bees and urban environments with our review findings.

**Review Articles analysed in comparison to our conducted review**	**Scope of study**	**Knowledge gaps / Research directions mentioned in the review article**	**What are the policy recommendations from the review articles?**	**Alignment/ agreement with our review findings presented in Sections 3 and 4**						
				Knowledge gaps in the review paper align with our findings<br><br>(alignment/ agreement with our findings presented in Section 3.1 & 4.3)	*How bees go about pollination and what knowledge is needed for (bees/) pollination (alignment/ agreement with our findings presented in Section 4)*	*On bee subtypes<br><br>(alignment/ agreement with our findings presented in Section 3.3)*	*How to perform research in the field of bee ecology*	*On how to perform research with regard to landscape characteristics<br><br>(alignment/ agreement with our findings presented in Section 4.1)*	*On the relation between (conservation) policy and bees<br><br>(alignment/agreement with our findings presented in Section 4.2)*	*New/ Other fields of research related to urban bees<br><br>(alignment/agreement with our findings presented in Section 4.3)*
Prendergast et al .(2022)	a comprehensive, semiquantitative review, involving 215 studies, on responses of bees to urban landscapes, and local and landscape variables proposed to influence bee abundance and diversity, globally	- Regions are not represented<br><br>- More specialized studies on single species are needed. Most single-species studies are on Bombus.<br><br>- Need for more historical land-use studies - Need for more long-term studies.<br><br>- Need for consistency and clarity on urbanization terms and natural landscapes<br><br>- Future research to focus on floral composition vs floral abundance and diversity.<br><br>- Future research to focus on bee metapopulation structure maintenance in urban landscapes	- Retain, restore, revegetate, and reconnect patches of remnant natural habitat throughout the urban matrix.<br><br>- Encourage homeowners, gardeners, landscape managers, and nurseries to focus on flowers that have been demonstrated to be visited by wild bees in the region. Flowers to be primarily native species and ensure oligolectic bee preferences are represented. Key exotic flowers that have a low risk of becoming invasive and offer quality and large volumes of nectar and pollen, especially outside the main flowering period.	☑	☑	☑	☑	☑	☐	☐
Rahimi et al. (2022)	A thematic review on the relationship between bees and urban agriculture, using 87 articles.	- the strategy of increasing the bee population in urban environments by establishing beehives is facing major challenges, and more studies are needed in this field.	- Recommendation to plant native plants with greater diversity for green roofs than sedum monoculture is recommended to attract bees<br>- For increasing floral resources in gardens, it is recommended to avoid planting flower species that need specialist bees for pollination.<br><br>- To attract bees, more attention on the habitat and nesting needs of above-ground nesting.<br><br>- Converting lawns into floral resources or carrying out agricultural activities around green spaces to be considered	☐	☑	☐	☐	☐	☐	☐
Brom et al (2022)	This review explores the potential role that urban landscapes can play in supporting pollinators in southern Africa.	- studies do not represent climatic regions evenly, and there is a gap in research from the African continent<br><br>- establish the value of wild and non-bee pollinators<br><br>- determining the nesting and habitat needs of non-bee species is, therefore, an important area for development in pollinator research, both for agriculture and urban landscapes. (not to be included because non-bee)	- Increase agricultural Local Habitat<br>Small patches of abundant flowers interspersed throughout the landscape<br>- Support and increase the flower community<br><br>- Targeted species/pollinator syndromes to support mutualisms (e.g. red and orange tubular flower for sunbirds)<br><br>- Produce attractive species lists of flowers for dissemination	☑	☐	☑	☐	☐	☐	☐
Buchholz & Egerer (2020)	systematically review the published literature on wild bee traits-urbanization relationships to synthesize the research findings regarding the relationship between functional traits and the amount of urbanization in the landscape surroundings	- urgent need for studies from Africa, Asia, and the Mediterranean region of Europe.<br><br>- future studies should increase the consistency of urban wild bee trait-based data collection, analysis, and transparency.<br><br>- future research should relate urban wild bee traits to ecosystem function and link traits to pollination of wild plant species along with cultural plants prevalent in cities (e.g. ornamental and food crops).	N/A	☑	☑	☑	☑	☐	☐	☐
Ferrari & Polidori (2022)	Relate city traits to bee diversity on a global scale.	- behavioral ecology of ground-nesting bees are still widely understudied<br><br>- ground-nesting bees are still widely understudied and further investigations are certainly needed	actions to improve the colonization of cities by diverse bee functional groups are certainly needed, particularly in the form of nature-based solutions, such as increasing richer flower strips and green spaces.	☐	☐	☑	☐	☐	☐	☐
Rahimi et al (2021).	we intend to present the results of various studies that have reported the efficiency of artificial nests in augmenting pollinators in a categorized manner.	No	No	☐	☐	☐	☐	☐	☐	☐

Table S.1 (continued). Juxtaposition of review articles on bees and urban environments with our review findings.

Review Articles analysed in comparison to our conducted review	Scope of study	Knowledge gaps / Research directions mentioned in the review article	What are the policy recommendations from the review articles?	Alignment/ agreement with our review findings presented in Sections 3 and 4						
				Knowledge gaps in the review paper align with our findings (alignment/ agreement with our findings presented in Section 3.1 & 4.3)	*How bees go about pollination and what knowledge is needed for (bees/) pollination (alignment/ agreement with our findings presented in Section 4)*	*On bee subtypes (alignment/ agreement with our findings presented in Section 3.3)*	*How to perform research in the field of bee ecology*	*On how to perform research with regard to landscape characteristics (alignment/ agreement with our findings presented in Section 4.1)*	*On the relation between (conservation) policy and bees (alignment/agreement with our findings presented in Section 4.2)*	*New/ Other fields of research related to urban bees (alignment/agreement with our findings presented in Section 4.3)*
Senapathi et al 2017	the interplay between landscape and pollinator communitiesm, including both natural and anthropogenic landscapes and all pollinators.	- More research on the effectiveness of management interventions for pollinators in urban areas, especially with respect to how habitats networks facilitate pollinators' dispersal across cities at the landscape scale and subsequent effects on population dynamics.<br><br>- future research to investigate how pollinator communities might respond to land-use change, particularly in the context of climate change	No	☐	☐	☐	☐	☑	☑	☑
Baldock 2020	To consider potential threats to animal pollinators in urban areas and conservation opportunities.	- Most studies to date have been in European or North American cities, so more research is needed to improve our understanding of urban pollinator communities in other regions where differences in the composition of urban areas may be important factors.<br><br>- In contrast, higher pollinator abundances were associated with higher-income neighborhoods in the UK. Further research is needed to understand these socio-economic drivers' effects and how they interact with other drivers.	- Ensuring that allotments and domestic gardens are protected in existing urban areas and included in future urban developments will, therefore, benefit pollinators and is likely to bring benefits for humans as well.<br><br>- Furthermore, increasing the areas of allotments and domestic gardens in urban areas is likely to lead to an increase in urban agriculture and therefore contribute to local food sustainability.<br><br>- Increasing the floral resources in public greenspace could be achieved by reducing mowing frequency or adding flowers	☑	☐	☐	☐	☑	☐	☑
Braman and Griffin, (2022)	Discuss case studies integrating elements of pest and pollinator management through plant selection, landscape and recreational area design, and community engagement with the goal of pollinator conservation.	- future research for better understanding of life cycles and pollination of non-bees. Understanding individual species impacts and their potential losses on plant populations is critical as individual functional contributions of species are dynamic, complex, and influenced by interspecific competitors.<br><br>- A better connection between biological sciences and social sciences could further conservation purposes	No	☐	☑	☑	☐	☐	☑	☑
Ayers and Rehan. (2021).	To summarize the<br><br>effectiveness of green spaces and other management and policy strategies that have been implemented in ameliorating pollinator losses and to detail the facets of urbanization that affect pollinating insect traits.	- Future research to examine the relationship between urban heat island and exotic bees<br><br>- Future longitudinal research about the urban landscape relationships with bees resilience<br><br>- Studies should be conducted to further substantiate the filtering out of pollinators that nest in the ground.	- Conservation initiatives within urban areas aimed at minimizing pollinator declines largely include the establishment and protection of green spaces throughout the urban matrix<br><br>- a reduction in intensive management practices such as excessive and frequent mowing not only benefits pollinators by preserving floral resources but also reduces costs associated with mowing - Improving upon plant and seed mix selections placed within green spaces could also enhance habitat quality and prevent ineffective spending.	☐	☑	☑	☐	☑	☐	☐

Hall et al 2017		- Future research to determine the relationships between bee diversity and patterns of residential land use across shrinking and growing cities<br>- Interdisciplinary research about legal, political, and institutional aspects regarding public land use, planting decisions, institutional policies, that affect actors' capacities to increase pollinator habitat also require further investigation.<br><br>- Future research to evaluate the effectiveness of pollinator seed mixes	No	☐	☑	☐	☐	☑	☑	☑

Table S.1 (continued). Juxtaposition of review articles on bees and urban environments with our review findings.

4,5	Scope of study	Knowledge gaps / Research directions mentioned in the review article	What are the policy recommendations from the review articles?	Alignment/ agreement with our review findings presented in Sections 3 and 4						
				Knowledge gaps in the review paper align with our findings<br><br>*(alignment/ agreement with our findings presented in Section 3.1 & 4.3)*	*How bees go about pollination and what knowledge is needed for (bees/) pollination (alignment/ agreement with our findings presented in Section 4)*	*On bee subtypes*<br><br>*(alignment/ agreement with our findings presented in Section 3.3)*	*How to perform research in the field of bee ecology*	*On how to perform research with regard to landscape characteristics*<br><br>*(alignment/ agreement with our findings presented in Section 4.1)*	*On the relation between (conservation) policy and bees*<br><br>*(alignment/agreement with our findings presented in Section 4.2)*	*New/ Other fields of research related to urban bees*<br><br>*(alignment/agreement with our findings presented in Section 4.3)*
Brant et al (2022).	The purpose of this paper is to provide a novel review of the important, albeit limited research on urban bee behavior, physiology, morphology, and genetics.	- more published urban bee studies from The United States, Canada, and Western Europe.<br>- broadening research efforts beyond honeybees and bumblebees will be an important next step in understanding urban bee disease ecology.<br>- no studies assessing bee forager patch use and patch leaving in urban systems.<br>- urban bees will exhibit increased frequency of direct, exploitative competitive behaviors for food resources compared to conspecifics living in more natural environments.<br>- in locations with neonicotinoid application, including urban farms and gardens, suburban lawns, and recreational green space such as golf courses, bee foraging vigor and efficiency will be lower across species compared to bee foragers in locations with no neonicotinoid use, and gene expression will likely be significantly different when compared to individuals residing in habitats with little to no xenobiotic application.<br>- urban plants provide less nutrient dense pollen than conspecifics in natural environments, leading to decreases in adult bee body size and overall bee health.	No	☑	☑	☑	☐	☐	☐	☑
Liang et al (2022)	Meta-analysis of multiple pollinators including bees.	- geography is limited to certain areas<br><br>- Research on pollinators is skewed towards bees.<br><br>- functional ecological studies of pollinators in cities are needed to investigate which traits influence their sensitivity to urbanization<br><br>- future research needs to disentangle the role of various biotic drivers (e.g. competition between native and non-native species, managed and wild species, predation and parasitism), as well as abiotic factors (e.g.<br><br>environmental stressors, landscape composition, urban green land-use type, size and connectivity, management practices) on pollinator assemblages in cities	No	☑	☐	☑	☐	☑	☐	☐

Wenzel et al (2020)	Thematic approach in a systematic review on all pollinators	- Abiotic factors such as heat and pollutants are hardly considered.<br><br>- Future research to fill the gap of hotspot areas such as Asia and Africa to support conservation efforts.<br><br>- configuration of the urban matrix and habitat isolation and their effects on pollinators (building height,<br><br>- There is much focus on pollinator diversity and little focus on pollination services.<br><br>- Barely any long-term studies were identified.	No	☑	☐	☐	☑	☑	☐	☐
